# Residual and Past Discrete Tsallis and Renyi Extropy with an Application to Softmax Function

**DOI:** 10.3390/e24121732

**Published:** 2022-11-27

**Authors:** Taghreed M. Jawa, Nahid Fatima, Neveen Sayed-Ahmed, Ramy Aldallal, Mohamed Said Mohamed

**Affiliations:** 1 Department of Mathematics and Statistics, College of Science, Taif University, P.O. Box 11099, Taif 21944, Saudi Arabia; 2 Department of Mathematics & Sciences, Prince Sultan University, Riyadh 11586, Saudi Arabia; 3 Department of Accounting, College of Business Administration in Hawtat Bani Tamim, Prince Sattam bin Abdulaziz University, Al-Kharj 11942, Saudi Arabia; 4 Department of Mathematics, Faculty of Education, Ain Shams University, Cairo 11341, Egypt

**Keywords:** extropy, Tsallis entropy, residual extropy, past extropy, residual Tsallis entropy, past Tsallis entropy, Renyi extropy, softmax function, ARIMA model

## Abstract

In this paper, based on the discrete lifetime distribution, the residual and past of the Tsallis and Renyi extropy are introduced as new measures of information. Moreover, some of their properties and their relation to other measures are discussed. Furthermore, an example of a uniform distribution of the obtained models is given. Moreover, the softmax function can be used as a discrete probability distribution function with a unity sum. Thus, applying those measures to the softmax function for simulated and real data is demonstrated. Besides, for real data, the softmax data are fit to a convenient ARIMA model.

## 1. Introduction

The entropy function has resulted in a substantial turn in the theory of information, such as the measure of uncertainty information. For a discrete random variable (R.V.) $X=\{x_{1},x_{2},\ldots ,x_{N}\}$, and p $=\{p_{1},p_{2},\ldots ,p_{N}\}$ is the corresponding probability vector, $p_{i}=P\left(X=x_{i}\right),$i=1,2,…,N. Thus, Shannon [1] produced the non-negative discrete entropy function as follows:

(1) $$S\left(p\right)=-\sum_{i=1}^{N}p_{i}\ln p_{i}.$$

Lad et al. [2] originated the extropy as a dual model of uncertainty. The non-negative extropy of the discrete R.V. X is given by

(2) $$ES\left(p\right)=-\sum_{i=1}^{N}\left(1-p_{i}\right)\ln\left(1-p_{i}\right).$$

Several measures of entropy and its generalization have been presented in the literature. Through the various generalizations of uncertainty, Tsallis [3] introduced the Tsallis entropy. The Tsallis entropy of the discrete R.V. X, 1≠α>0, is given by

(3) $$T_{\alpha }\left(p\right)=\frac{1}{\alpha -1}\left(1-\sum_{i=1}^{N}p_{i}^{\alpha }\right)=\frac{1}{\alpha -1}\left(\sum_{i=1}^{N}p_{i}-\sum_{i=1}^{N}p_{i}^{\alpha }\right),$$

when α is 1, then $\lim_{\alpha \to 1}T_{\alpha }\left(p\right)=S\left(p\right)$.

Recently, Xue and Deng [4] proposed the measure Tsallis extropy, as a complementary dual of the Tsallis uncertainty function and studied its maximum value. Then, Balakrishnan et al. [5] studied the Tsallis extropy and applied it to pattern recognition. The Tsallis extropy of the discrete R.V. X, 1≠α>0, is given by

(4) $$TES_{\alpha }\left(p\right)=\frac{1}{\alpha -1}\left(\sum_{i=1}^{N}\left(1-p_{i}\right)-\sum_{i=1}^{N}{\left(1-p_{i}\right)}^{\alpha }\right)=\frac{1}{\alpha -1}\left(N-1-\sum_{i=1}^{N}{\left(1-p_{i}\right)}^{\alpha }\right),$$

when α is 1, then $\lim_{\alpha \to 1}TES_{\alpha }\left(p\right)=ES_{\alpha }\left(p\right)$.

Based on the continuous lifetime distribution, Ebrahimi [6] discussed the measure of the entropy of the residual lifetime distribution. Furthermore, Di Crescenzo and Longobardi [7] presented the measure of the entropy of the past lifetime distribution.

Based on the discrete lifetime distribution, Gao and Shang [8] developed the generalized past entropy via the grain exponent based on oscillations. Li and Shang [9] introduced the modified discrete generalized past entropy via the grain exponent.

The innovation of this paper lies in presenting the residual and past versions of the Tsallis and Renyi extropy based on the discrete lifetime distribution. Moreover, we apply those models to the softmax function for simulated and real data. Besides, we discuss the residuals of the softmax real data and give a suitable ARIMA model to fit the data. The remaining article is structured as follows: Section 2 gives the suggested models with their properties and relation to other measures. Besides, we give an example of the models for a uniform distribution. Section 3 applies the softmax function to the suggested models on simulated and real data. Furthermore, we discuss the residuals and the fit ARIMA model to the softmax U.S. consumption data. Finally, Section 4 finishes the article with some conclusions.

## 2. The Suggested Models

Extropy is a favorite instrument to enhance the Tsallis entropy, which is the dual model of information entropy. The Tsallis extropy combines the advantages of the Tsallis entropy and extropy. In this section, we present the residual and past versions of the Tsallis extropy based on the discrete lifetime distribution.

Let the discrete R.V. X supported with $S=\{x_{1},x_{2},\ldots ,x_{N}\}$, and the corresponding probability vector $p=\{p_{1},p_{2},\ldots ,p_{N}\}$. Then, the residual and past discrete Tsallis entropy, 1≠α>0, is given, respectively, by

(5) $$RT_{\alpha }\left(p;t\right)=\frac{1}{\alpha -1}\left(1-\sum_{i=t}^{N}{\left(\frac{p_{i}}{{\bar{P}}_{t}}\right)}^{\alpha }\right)=\frac{1}{\alpha -1}\left(\sum_{i=1}^{N}p_{i}-\sum_{i=t}^{N}{\left(\frac{p_{i}}{{\bar{P}}_{t}}\right)}^{\alpha }\right),$$

(6) $$PT_{\alpha }\left(p;t\right)=\frac{1}{\alpha -1}\left(1-\sum_{i=1}^{t}{\left(\frac{p_{i}}{P_{t}}\right)}^{\alpha }\right)=\frac{1}{\alpha -1}\left(\sum_{i=1}^{N}p_{i}-\sum_{i=1}^{t}{\left(\frac{p_{i}}{P_{t}}\right)}^{\alpha }\right),$$

where $P_{t}=P\left(X\leq t\right)=\sum_{i=1}^{t}p_{i}$ and ${\bar{P}}_{t}=1-P_{t}$, and the interceptive parameter *t* is between 1 and N. Moreover, the residual and past discrete extropy is given, respectively, by

(7) $$RES\left(p;t\right)=-\sum_{i=t}^{N}\left(1-\frac{p_{i}}{{\bar{P}}_{t}}\right)\ln\left(1-\frac{p_{i}}{{\bar{P}}_{t}}\right),$$

(8) $$PES\left(p;t\right)=-\sum_{i=1}^{t}\left(1-\frac{p_{i}}{P_{t}}\right)\ln\left(1-\frac{p_{i}}{P_{t}}\right).$$

Motivated by the concept of the Tsallis extropy and the discrete lifetime distribution, we introduce the residual and past of the Tsallis extropy, respectively, as follows:

(9) $$RTES_{\alpha }\left(p;t\right)=\frac{1}{\alpha -1}\left(\sum_{i=1}^{N}\left(1-p_{i}\right)-\sum_{i=t}^{N}{\left(1-\frac{p_{i}}{{\bar{P}}_{t}}\right)}^{\alpha }\right)=\frac{1}{\alpha -1}\left(N-1-\sum_{i=t}^{N}{\left(1-\frac{p_{i}}{{\bar{P}}_{t}}\right)}^{\alpha }\right),$$

(10) $$PTES_{\alpha }\left(p;t\right)=\frac{1}{\alpha -1}\left(\sum_{i=1}^{N}\left(1-p_{i}\right)-\sum_{i=1}^{t}{\left(1-\frac{p_{i}}{P_{t}}\right)}^{\alpha }\right)=\frac{1}{\alpha -1}\left(N-1-\sum_{i=1}^{t}{\left(1-\frac{p_{i}}{P_{t}}\right)}^{\alpha }\right).$$

In the following proposition, we illustrate the past and residual Tsallis extropy behavior according to the value of α.

**Proposition** **1.** *Let the discrete R.V. X supported with $S=\{x_{1},x_{2},\ldots ,x_{N}\}$, and the corresponding probability vector $p=\{p_{1},p_{2},\ldots ,p_{N}\}$. Then, from the past and residual Tsallis extropy given in Equations (10) and (9), respectively, we have:*

*1.*  *The past Tsallis extropy is positive (negative) if α>1 (0<α<1).*
*2.*  *The residual Tsallis extropy is positive (negative) if α>1 (0<α<1).*

**Proof.** 

1. The past Tsallis extropy given in Equation (10) can be rewritten as follows:
  $$\begin{array}{cc} PTES_{\alpha }\left(p;t\right) & =\frac{1}{\alpha -1}\left(\sum_{i=1}^{N}\left(1-p_{i}\right)-\sum_{i=1}^{t}{\left(1-\frac{p_{i}}{P_{t}}\right)}^{\alpha }\right)\\ & =\frac{1}{\alpha -1}\left(\sum_{i=1}^{N}\left(1-P_{t}+P_{t}-p_{i}\right)-\sum_{i=1}^{t}{\left(1-\frac{p_{i}}{P_{t}}\right)}^{\alpha }\right)\\ & =\frac{1}{\alpha -1}\left(N\left(1-P_{t}\right)+\sum_{i=t+1}^{N}\left(P_{t}-p_{i}\right)+\sum_{i=1}^{t}\left(P_{t}-p_{i}\right)\left(1-\frac{1}{P_{t}}{\left(1-\frac{p_{i}}{P_{t}}\right)}^{\alpha -1}\right)\right). \end{array}$$
  Moreover, we have that the term $N(1-P_{t})\geq 0$ is greater than the other two terms, and $P_{t}\geq p_{i},i=1,2,\ldots ,N$. Then, the proof is obtained.
2. The residual Tsallis extropy given in Equation (9) can be rewritten as follows:
  $$\begin{array}{cc} RTES_{\alpha }\left(p;t\right) & =\frac{1}{\alpha -1}\left(\sum_{i=1}^{N}\left(1-{\bar{P}}_{t}+{\bar{P}}_{t}-p_{i}\right)-\sum_{i=t}^{N}{\left(1-\frac{p_{i}}{{\bar{P}}_{t}}\right)}^{\alpha }\right)\\ & =\frac{1}{\alpha -1}\left(N\left(1-{\bar{P}}_{t}\right)+\sum_{i=1}^{t-1}\left({\bar{P}}_{t}-p_{i}\right)+\sum_{i=t}^{N}\left({\bar{P}}_{t}-p_{i}\right)\left(1-\frac{1}{{\bar{P}}_{t}}{\left(1-\frac{p_{i}}{{\bar{P}}_{t}}\right)}^{\alpha -1}\right)\right). \end{array}$$
  Moreover, the proof is obtained similarly.

□

**Example** **1.** *Suppose that the discrete R.V. X has a uniform distribution over {1,…,N}. Then, the residual and past Tsallis extropy are given, respectively, by*

$$\begin{array}{cc} RTES_{\alpha }\left(p;t\right) & =\frac{1}{\alpha -1}\left(N-1-\sum_{i=t}^{N}{\left(1-\frac{1}{N-t}\right)}^{\alpha }\right)\\ & =\frac{1}{\alpha -1}\left(N-1-\left(N-t+1\right){\left(1-\frac{1}{N-t}\right)}^{\alpha }\right). \end{array}$$

$$\begin{array}{cc} PTES_{\alpha }\left(p;t\right) & =\frac{1}{\alpha -1}\left(N-1-\sum_{i=1}^{t}{\left(1-\frac{1}{t}\right)}^{\alpha }\right)\\ & =\frac{1}{\alpha -1}\left(N-1-t{\left(1-\frac{1}{t}\right)}^{\alpha }\right). \end{array}$$

In the following proposition, we will establish the relation between the past and residual Tsallis extropy and the past and residual extropy.

**Proposition** **2.** *Suppose that the discrete R.V. X has finite support with corresponding probability vector $p=\{p_{1},p_{2},\ldots ,p_{N}\}$. Then, from the past and residual Tsallis extropy given in Equations (10) and (9), respectively, and the past and residual extropy given in Equations (8) and (7), respectively, we have:*

*1.*  *From Equations (10) and (8), we have*
$$\lim_{\alpha \to 1}PTES_{\alpha }\left(p;t\right)=PES\left(p;t\right).$$
*2.*  *From Equations (9) and (7), we have*
$$\lim_{\alpha \to 1}RTES_{\alpha }\left(p;t\right)=RES\left(p;t\right).$$

**Proof.** 

1. From (10) and applying $L^{\prime }H\hat{o}pital^{\prime }s$ rule, we have
  $$\begin{array}{cc} \lim_{\alpha \to 1}PTES_{\alpha }\left(p;t\right) & =\lim_{\alpha \to 1}\frac{1}{\alpha -1}\left(N-1-\sum_{i=1}^{t}{\left(1-\frac{p_{i}}{P_{t}}\right)}^{\alpha }\right)\\ \; & =\lim_{\alpha \to 1}-\sum_{i=1}^{t}{\left(1-\frac{p_{i}}{P_{t}}\right)}^{\alpha }\ln\left(1-\frac{p_{i}}{P_{t}}\right).\\ \; & =-\sum_{i=1}^{t}\left(1-\frac{p_{i}}{P_{t}}\right)\ln\left(1-\frac{p_{i}}{P_{t}}\right)\\ \; & =PES(p;t). \end{array}$$
2. From (9) and applying $L^{\prime }H\hat{o}pital^{\prime }s$ rule, we have
  $$\begin{array}{cc} \lim_{\alpha \to 1}RTES_{\alpha }\left(p;t\right) & =\lim_{\alpha \to 1}\frac{1}{\alpha -1}\left(N-1-\sum_{i=t}^{N}{\left(1-\frac{p_{i}}{{\bar{P}}_{t}}\right)}^{\alpha }\right)\\ \; & =\lim_{\alpha \to 1}-\sum_{i=t}^{N}{\left(1-\frac{p_{i}}{{\bar{P}}_{t}}\right)}^{\alpha }\ln\left(1-\frac{p_{i}}{{\bar{P}}_{t}}\right).\\ \; & =-\sum_{i=t}^{N}\left(1-\frac{p_{i}}{{\bar{P}}_{t}}\right)\ln\left(1-\frac{p_{i}}{{\bar{P}}_{t}}\right)\\ \; & =RES(p;t). \end{array}$$

□

In the next proposition, we will obtain the relation between the past and residual Tsallis extropy and the past and residual Tsallis entropy when the choice of the parameter α=2.

**Proposition** **3.** *Suppose that the discrete R.V. X has finite support with the corresponding probability vector $p=\{p_{1},p_{2},\ldots ,p_{N}\}$. Then, from the past and residual Tsallis extropy given in Equations (10) and (9), respectively, and the past and residual Tsallis entropy given in Equations (6) and (5), respectively, we have:*

*1.*  *From Equations (10) and (6), we have*
$$PTES_{2}\left(p;t\right)=N-t+PT_{2}\left(p;t\right),$$
  *if t=N, then $PTES_{2}\left(p;t\right)=PT_{2}\left(p;t\right)$.*
*2.*  *From Equations (9) and (5), we have*
$$RTES_{2}\left(p;t\right)=t-1+RT_{2}\left(p;t\right),$$
  *if t=1, then $RTES_{2}\left(p;t\right)=RT_{2}\left(p;t\right)$.*

**Proof.** 

1. From (10), when α=2, we have
  $$\begin{array}{cc} PTES_{2}\left(p;t\right) & =\frac{1}{2-1}\left(N-1-\sum_{i=1}^{t}{\left(1-\frac{p_{i}}{P_{t}}\right)}^{2}\right)\\ & =N-1-\sum_{i=1}^{t}\left(1-2\frac{p_{i}}{P_{t}}+{\left(\frac{p_{i}}{P_{t}}\right)}^{2}\right).\\ & =N-t+1-\sum_{i=1}^{t}{\left(\frac{p_{i}}{P_{t}}\right)}^{2}\\ & =N-t+PT_{2}\left(p;t\right). \end{array}$$
2. From (9), when α=2, we have
  $$\begin{array}{cc} RTES_{2}\left(p;t\right) & =\frac{1}{2-1}\left(N-1-\sum_{i=t}^{N}{\left(1-\frac{p_{i}}{{\bar{P}}_{t}}\right)}^{2}\right)\\ & =N-1-\sum_{i=t}^{N}\left(1-2\frac{p_{i}}{{\bar{P}}_{t}}+{\left(\frac{p_{i}}{{\bar{P}}_{t}}\right)}^{2}\right)\\ & =t-1+1-\sum_{i=t}^{N}{\left(\frac{p_{i}}{{\bar{P}}_{t}}\right)}^{2}\\ & =t-1+RT_{2}\left(p;t\right). \end{array}$$

□

In the following theorem, we will show the relation between the past and residual Tsallis extropy and past and residual Tsallis entropy according to the value of α.

**Theorem** **1.** *Assume that the discrete R.V. X has finite support $\left\{x_{1},x_{2},\ldots ,x_{N}\right\}$ with the corresponding probability vector $p=\{p_{1},p_{2},\ldots ,p_{N}\}$. Then, from the past and residual Tsallis extropy given in Equations (10) and (9), respectively, and the past and residual Tsallis entropy given in Equations (6) and (5), respectively, we have:*

*1.*  *For 0<α<1, we obtain*
$$PT_{\alpha }\left(p;t\right)>PTES_{\alpha }\left(p;t\right),\;and\;RT_{\alpha }\left(p;t\right)>RTES_{\alpha }\left(p;t\right).$$
*2.*  *For α>1, we obtain*
$$PT_{\alpha }\left(p;t\right)<PTES_{\alpha }\left(p;t\right),\;and\;RT_{\alpha }\left(p;t\right)<RTES_{\alpha }\left(p;t\right).$$

**Proof.**  From Equations (9) and (5), we have

$$\begin{array}{cc} \frac{PT_{\alpha }\left(p;t\right)}{PTES_{\alpha }\left(p;t\right)} & =\frac{1-\sum_{i=1}^{t}{\left(\frac{p_{i}}{P_{t}}\right)}^{\alpha }}{N-1-\sum_{i=1}^{t}{\left(1-\frac{p_{i}}{P_{t}}\right)}^{\alpha }}\\ & =\frac{P_{t}^{\alpha }-\sum_{i=1}^{t}p_{i}^{\alpha }}{\left(N-1\right)P_{t}^{\alpha }-\sum_{i=1}^{t}{\left(P_{t}-p_{i}\right)}^{\alpha }}\\ & =\frac{{\left(\sum_{i=1}^{t}p_{i}\right)}^{\alpha }-\sum_{i=1}^{t}p_{i}^{\alpha }}{\left(N-1\right){\left(\sum_{i=1}^{t}p_{i}\right)}^{\alpha }-\sum_{i=1}^{t}{\left(\left(\sum_{i=1}^{t}p_{i}\right)-p_{i}\right)}^{\alpha }}. \end{array}$$

It can be simply noted that: if 0<α<1, then ${\left(\sum_{i=1}^{t}p_{i}\right)}^{\alpha }<\sum_{i=1}^{t}p_{i}^{\alpha }$, and if α>1, then ${\left(\sum_{i=1}^{t}p_{i}\right)}^{\alpha }>\sum_{i=1}^{t}p_{i}^{\alpha }$. Moreover, we have $\left(N-1\right)P_{t}^{\alpha }>P_{t}^{\alpha }-\sum_{i=1}^{t}p_{i}^{\alpha }>-\sum_{i=1}^{t}{\left(P_{t}-p_{i}\right)}^{\alpha }$, ∀α≠1. In the sequel, the results follow. □

### Residual and Past Discrete Renyi Extropy

In the same manner, we can discuss the residual and past discrete Renyi extropy. Let the discrete R.V. X supported with $S=\{x_{1},x_{2},\ldots ,x_{N}\}$, and the corresponding probability vector $p=\{p_{1},p_{2},\ldots ,p_{N}\}$. Then, the Renyi extropy was introduced by Liu and Xiao [10] as follows:

(11) $$\begin{array}{cc} RES_{\alpha }\left(p;t\right) & =\frac{1}{1-\alpha }\left(-\left(N-1\right)\ln\left(N-1\right)+\left(N-1\right)\ln\left(\sum_{i=1}^{N}{\left(1-p_{i}\right)}^{\alpha }\right)\right),\;1\neq \alpha >0. \end{array}$$

Motivated by the concept of the Renyi extropy and the discrete lifetime distribution, we introduce the residual and past of the Renyi extropy, respectively, as follows:

(12) $$RRES_{\alpha }\left(p;t\right)=\frac{1}{1-\alpha }\left(-\left(N-t\right)\ln\left(N-t\right)+\left(N-t\right)\ln\left(\sum_{i=t}^{N}{\left(1-\frac{p_{i}}{{\bar{P}}_{t}}\right)}^{\alpha }\right)\right),$$

(13) $$PRES_{\alpha }\left(p;t\right)=\frac{1}{1-\alpha }\left(-\left(t-1\right)\ln\left(t-1\right)+\left(t-1\right)\ln\left(\sum_{i=1}^{t}{\left(1-\frac{p_{i}}{P_{t}}\right)}^{\alpha }\right)\right).$$

In the following proposition, we will show the relation between the past and residual Renyi extropy and the past and residual extropy.

**Proposition** **4.** *Assume that the discrete R.V. X has finite support with the corresponding probability vector $p=\{p_{1},p_{2},\ldots ,p_{N}\}$. Then, from the past and residual Renyi extropy given in Equations (13) and (12), respectively, and the past and residual extropy given in Equations (8) and (7), respectively, we have:*

*1.*  *From Equations (13) and (8), we have*
$$\lim_{\alpha \to 1}PRES_{\alpha }\left(p;t\right)=PES\left(p;t\right).$$
*2.*  *From Equations (12) and (7), we have*
$$\lim_{\alpha \to 1}RRES_{\alpha }\left(p;t\right)=RES\left(p;t\right).$$

**Example** **2.** *Suppose that the discrete R.V. X has a uniform distribution over {1,…,N}. Then, the residual and past Renyi extropy are given, respectively, by*

$$\begin{array}{cc} RRES_{\alpha }\left(p;t\right) & =\frac{1}{\alpha -1}\left(-\left(N-t\right)\left(\ln\left(N-t\right)-\ln\left({\left(\frac{-1+N-t}{N-t}\right)}^{\alpha }\left(1+N-t\right)\right)\right)\right). \end{array}$$

$$\begin{array}{cc} PRES_{\alpha }\left(p;t\right) & =\frac{1}{\alpha -1}\left(-\left(-1+t\right)\left(\ln\left(-1+t\right)-t\ln{\left(\frac{-1+t}{t}\right)}^{\alpha }\right)\right). \end{array}$$

## 3. Applications

In this section, we will use different data sources and the softmax function to obtain the corresponding probability vector p, then discuss the behavior of the residual and past Tsallis extropy.

### 3.1. Softmax Function

It has been noted in the real world that the actual R.V. may be continuous in disposition, but discrete when we observe it, for example in a hospital, the number of days a patient stays or a patient survives after treatment. Accordingly, it is appropriate to fit these positions by discrete distributions developed from continuous distributions. One of the most well-known functions in engineering and science is the softmax function. It has usage in many fields such as game theory [11,12,13], reinforcement learning [14], and machine learning [15,16]. This normalized exponential function is used to transform a vector $y={\left(y_{1},y_{2},\ldots ,y_{N}\right)}^{t}\in R^{N}$ into a unit sum vector $\in {\left(0,1\right)}^{N}$ as follows

(14) $$\begin{array}{cc} SMAX{\left(y\right)}_{i} & =\frac{e^{y_{i}}}{\sum_{l=1}^{N}e^{y_{l}}}\\ & =e^{y-\log\left(\sum_{l=1}^{N}e^{y_{l}}\right)},\;i=1,2,\ldots ,N. \end{array}$$

#### 3.1.1. Standard Normal Distribution

**Figure 1:**
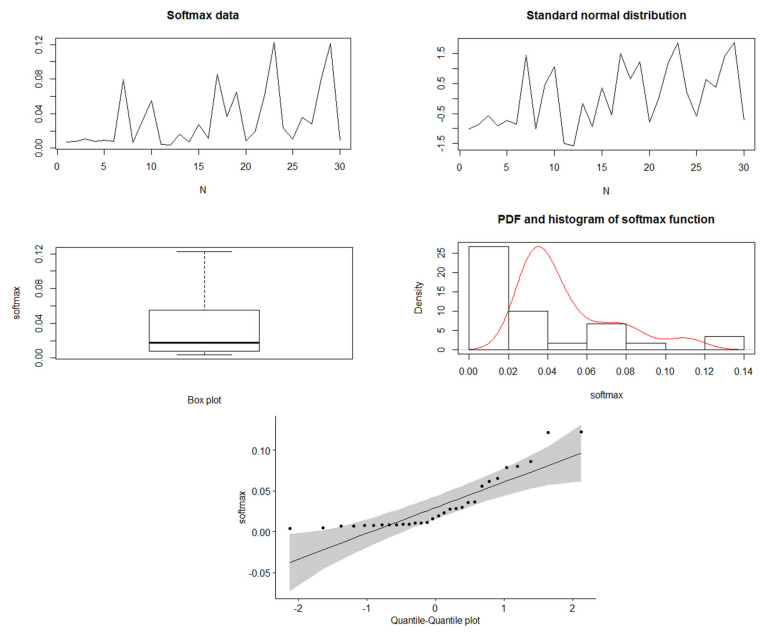
Simulated data of N(0,1) and the softmax data, N=30.

**Figure 2:**
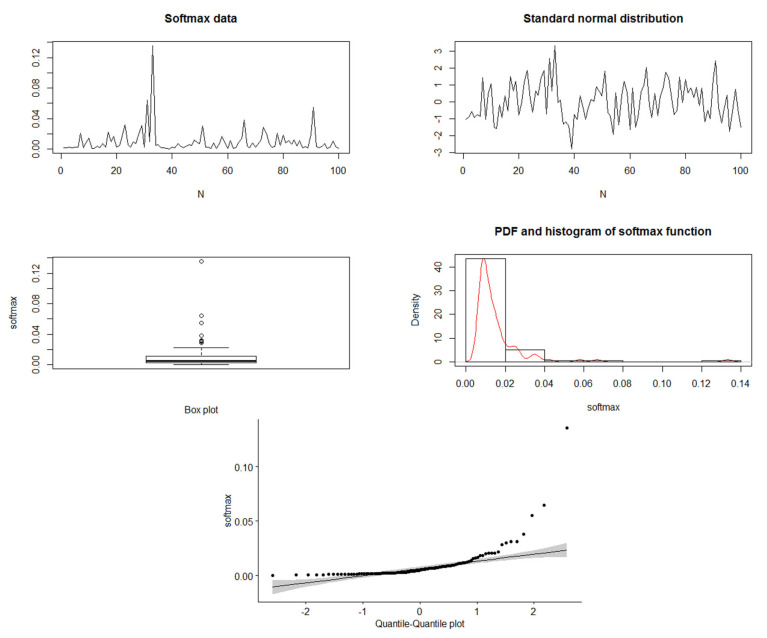
Simulated data of N(0,1) and the softmax data, N=100.

**Figure 3:**
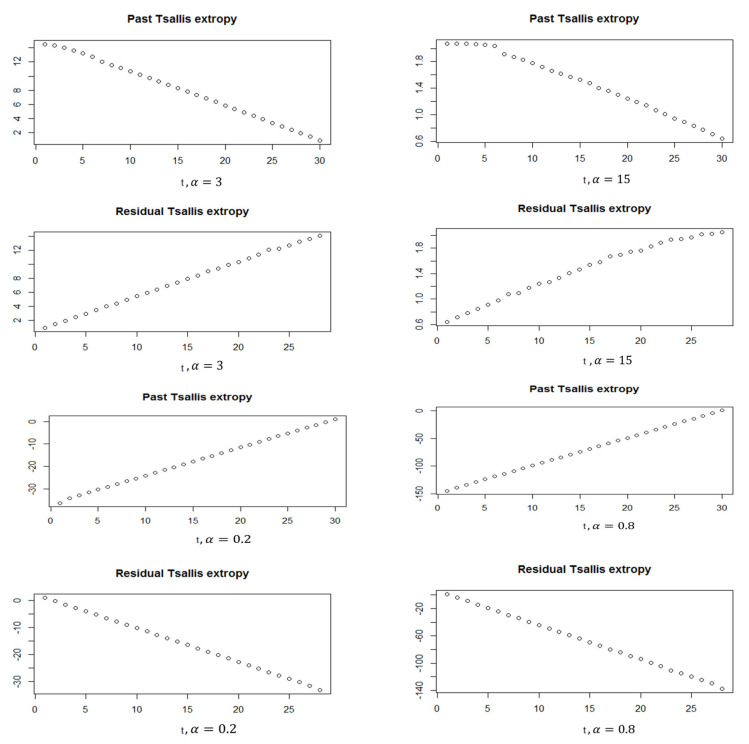
Past and residual Tsallis extropy, N=30, α=0.2,0.8,3,15.

**Figure 4:**
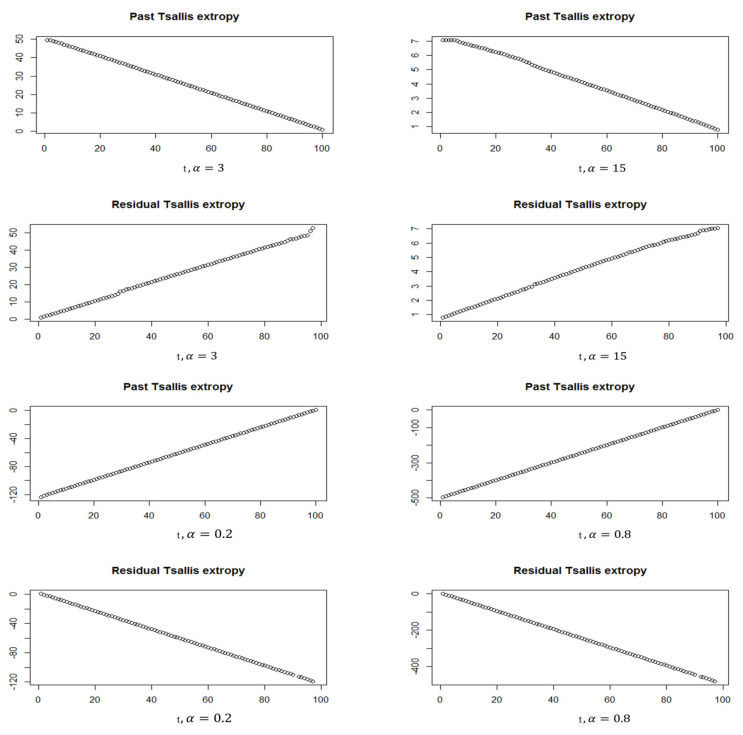
Past and residual Tsallis extropy, N=100, α=0.2,0.8,3,15.

In this part, we generate data from standard normal distribution (N(0,1)) and use the softmax function to obtain the corresponding probability vector with a unity sum. Figure 1 and Figure 2 show the simulated data of N(0,1), besides the softmax data when N = 30 and 100, respectively. For the softmax data when N=30, we see the mean = 0.0333 and the variance = 0.001195, and it shows the quantile–quantile plot of the softmax data, which can be noted to be far from normality. When N=100, we see that the mean = 0.01 and the variance = 0.0002753. Therefore, we can see that the variance is decreasing by increasing the sample. Furthermore, in the quantile–quantile plot of the softmax data, it starts to be closer to normality, but still, the extremely standardized residuals (on both ends) are more extensive than they would be for normal data. Moreover, Figure 3 and Figure 4 show the past and residual Tsallis extropy when α=0.2,0.8,3,15, which can be noted as follows: By increasing t, the past Tsallis extropy decreases (increases) for α>1(0<α<1). Furthermore, the residual Tsallis extropy increases (decreases) for α>1(0<α<1).

#### 3.1.2. Real Data: U.S. Consumption and Personal Income Quarterly Changes

**Figure 5:**
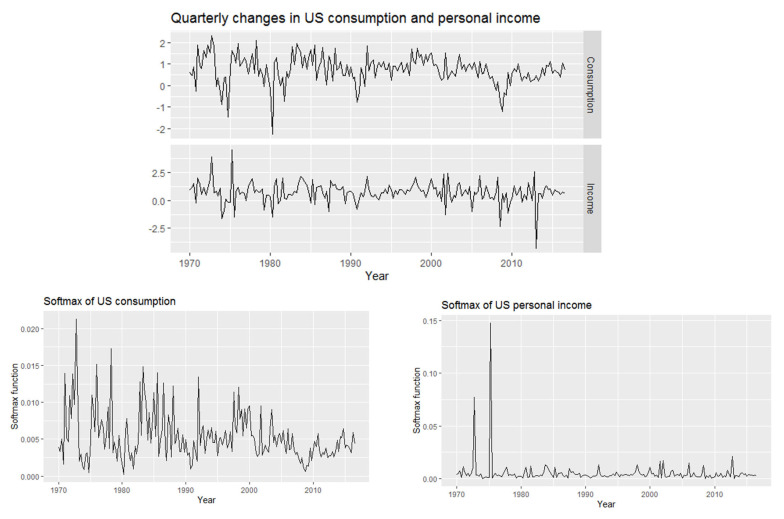
Quarterly changes in U.S. consumption and personal income data from 1970 to 2016 and the corresponding softmax data.

**Figure 6:**
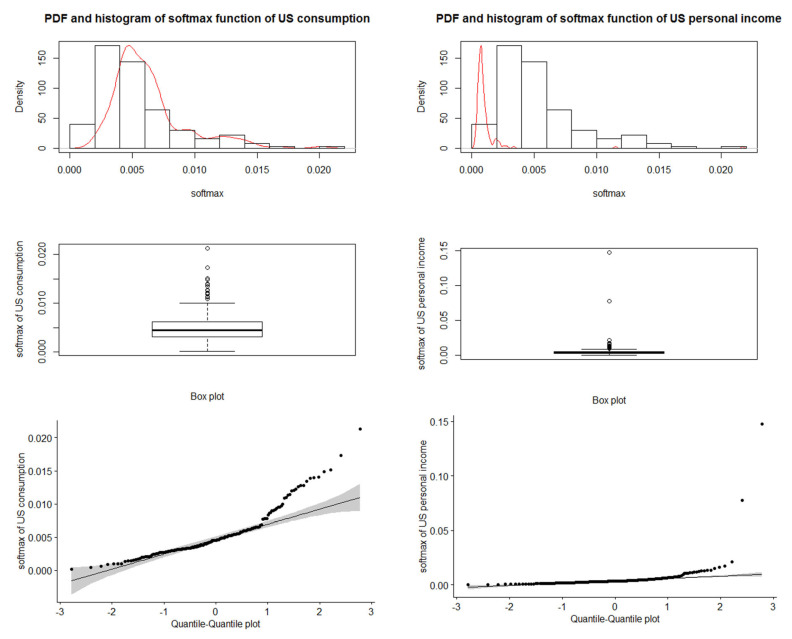
Data analysis of softmax U.S. consumption (**left** panel) and softmax U.S. personal income (**right** panel).

This part presents the quarterly changes in U.S. consumption and personal income data from 1970 to 2016. First, we want to discuss the softmax data obtained from our data. Figure 5 displays the U.S. consumption and income data and the corresponding softmax data. Figure 6 shows the data analysis of the softmax U.S. consumption and personal income, such that the mean of softmax U.S. consumption is 0.00534759 and the variance is 0.000011557 and the mean softmax of U.S. personal income is 0.00534759 and the variance is 0.0000149023. Moreover, the extremely standardized residuals (on both ends) are larger than they would be for normal data.

**Figure 7:**
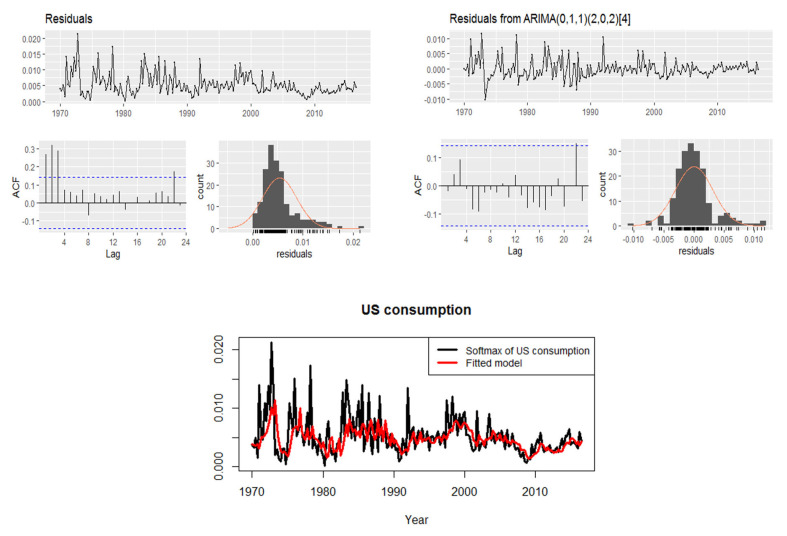
Residuals of softmax U.S. consumption and its fit model $ARIMA\left(0,1,1\right){\left(2,0,2\right)}_{4}$.

In Figure 7, based on the softmax U.S. consumption data, we see the residuals of the data before fitting them to a preferred model. Therefore, there are significant spikes in the ACF according to its residuals. Then, our aim now is to find a suitable model based on the ACF. Correspondingly, Figure 7 shows the residuals of the model fit to the data. Thus, the best model that fits the data is $ARIMA\left(0,1,1\right){\left(2,0,2\right)}_{4}$ with *p*-value = 0.1502, AIC = 350.51, and it accepts the Ljung–Box test. Accordingly, all the spikes are within the significant ACF limits, except for a single spike that exceeds a small limit. Note that the general form of a seasonal ARIMA model is $ARIMA\left(p,b,q\right){\left(p,b,q\right)}_{s}$, where ${\left(p,b,q\right)}_{s}$ and (p,b,q) are the seasonal and non-seasonal part of of the model, respectively, and *s* is the number of observations per year.

**Figure 8:**
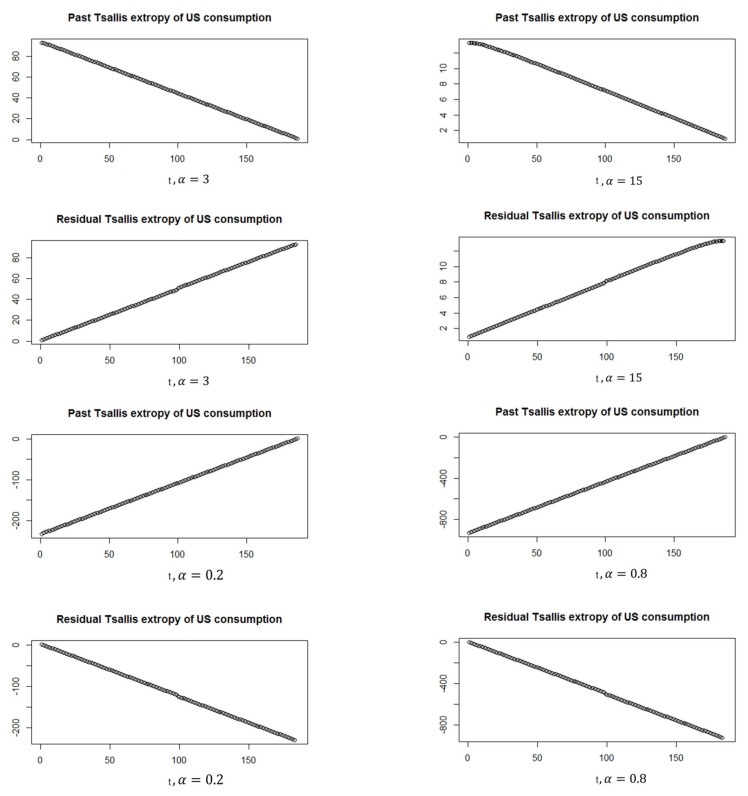
Past and residual Tsallis extropy of softmax U.S. consumption.

Moreover, Figure 8 shows the past and residual Tsallis extropy of the softmax U.S. consumption data, which can be noted as follows: By increasing t, the past Tsallis extropy decreases (increases) for α>1 (0<α<1). Furthermore, the residual Tsallis extropy increases (decreases) for α>1 (0<α<1).

## 4. Conclusions

In this article, we offered the residual and past discrete Tsallis and Renyi extropy. Moreover, we discussed some properties and relations of the residual and past discrete Tsallis entropy with respect to our models. Moreover, we chose the softmax function as a discrete probability distribution function and studied its behavior for simulated and real data. We discussed the residuals of the softmax data for real data and suggested that the appropriate ARIMA model fits the data. Furthermore, we obtained the past and residual Tsallis extropy and studied its increasing and decreasing according to the value of α. Finally, we can apply those models to ordered variables and their concomitants for future work. For more details, see [17,18,19]. For applications, see [20,21,22].

## Data Availability

The simulated data used to support the findings of this study are included within the article.
